# A Meningococcal Outer Membrane Vesicle Vaccine with Overexpressed Mutant FHbp Elicits Higher Protective Antibody Responses in Infant Rhesus Macaques than a Licensed Serogroup B Vaccine

**DOI:** 10.1128/mBio.01231-19

**Published:** 2019-06-18

**Authors:** Peter T. Beernink, Vianca Vianzon, Lisa A. Lewis, Gregory R. Moe, Dan M. Granoff

**Affiliations:** a Center for Immunobiology and Vaccine Development, University of California, San Francisco (UCSF) Benioff Children’s Hospital Oakland, Oakland, California, USA; b Department of Pediatrics, School of Medicine, UCSF, San Francisco, California, USA; c Division of Immunology and Infectious Diseases, University of Massachusetts Medical School, Worcester, Massachusetts, USA; Albert Einstein College of Medicine; Boston University; National Institute for Biological Standards and Control

**Keywords:** factor H binding protein, *Neisseria meningitidis*, outer membrane vesicles, nonhuman primate, vaccines

## Abstract

MenB-4C (Bexsero; GlaxoSmithKline Biologicals) is a licensed meningococcal vaccine for capsular B strains. The vaccine contains detergent-extracted outer membrane vesicles (dOMV) and three recombinant proteins, of which one is factor H binding protein (FHbp). In previous studies, overexpression of FHbp in native OMV (NOMV) with genetically attenuated endotoxin (LpxL1) and/or by the use of mutant FHbp antigens with low factor H (FH) binding increased serum bactericidal antibody (SBA) responses. In this study, we immunized 13 infant macaques with 2 doses of NOMV with overexpressed mutant (R41S) FHbp with low binding to macaque FH (NOMV-FHbp). Control macaques received MenB-4C (*n* = 13) or aluminum hydroxide adjuvant alone (*n* = 4). NOMV-FHbp elicited a 2-fold higher IgG anti-FHbp geometric mean titer (GMT) than MenB-4C (*P = *0.003), and the anti-FHbp repertoire inhibited binding of FH to FHbp, whereas anti-FHbp antibodies to MenB-4C enhanced FH binding. MenB-4C elicited a 10-fold higher GMT against strain NZ98/254, which was used to prepare the dOMV component, whereas NOMV-FHbp elicited an 8-fold higher GMT against strain H44/76, which was the parent of the mutant NOMV-FHbp vaccine strain. Against four strains with PorA mismatched to both of the vaccines and different FHbp sequence variants, NOMV-FHbp elicited 6- to 14-fold higher SBA GMTs than MenB-4C (*P *≤* *0.0002). Two of 13 macaques immunized with MenB-4C but 0 of 17 macaques immunized with NOMV-FHbp or adjuvant developed serum anti-FH autoantibodies (*P = *0.18). Thus, the mutant NOMV-FHbp approach has the potential to elicit higher and broader SBA responses than a licensed group B vaccine that contains wild-type FHbp that binds FH. The mutant NOMV-FHbp also might pose less of a risk of eliciting anti-FH autoantibodies.

## Abstract (precis)

There are two licensed meningococcal capsular B vaccines. Both contain recombinant factor H binding protein (FHbp), which can bind to host complement factor H (FH). The limitations of these vaccines include a lack of protection against some meningococcal strains and the potential to elicit autoantibodies to FH. We immunized infant macaques with a native outer membrane vesicle (NOMV) vaccine with genetically attenuated endotoxin and overproduced mutant FHbp with low binding to FH. The NOMV-FHbp vaccine stimulated higher levels of protective serum antibodies than a licensed meningococcal group B vaccine against five of six genetically diverse meningococcal strains tested. Two of 13 macaques immunized with the licensed vaccine, which contains FHbp that binds macaque FH, but 0 of 17 macaques given NOMV-FHbp or the negative control developed serum anti-FH autoantibodies Thus, in a relevant nonhuman primate model, the NOMV-FHbp vaccine elicited greater protective antibodies than the licensed vaccine and may pose less of a risk of anti-FH autoantibody.

## INTRODUCTION

Two meningococcal serogroup B vaccines are licensed in North America and Europe. MenB-4C (Bexsero; GlaxoSmithKline Biologicals) contains four components: three are recombinant proteins, one of which is factor H binding protein (FHbp), and the fourth is detergent-extracted outer membrane vesicles (dOMV) (1). The detergent treatment is used to decrease endotoxin activity but also removes desirable detergent-soluble antigens, such as FHbp (2). The second vaccine, MenB-FHbp (Trumenba; Pfizer), contains two recombinant, lipidated FHbp variants (3), one from each of the two phylogenic subfamilies (4, 5). Both vaccines elicit human complement-mediated serum bactericidal activity (SBA), which correlates with protection against developing meningococcal disease. However, for both vaccines, SBA titers are low against some strains (6, 7) and titers can fall below protective levels within 12 months (6–8). Also, the FHbp antigens in both vaccines bind to host factor H (FH), which in experimental animal models decreased the protective antibody responses to FHbp (9, 10) and stimulated anti-FH autoantibodies (10, 11). Therefore, there is a need for an expanded-spectrum meningococcal group B vaccine that is capable of eliciting higher SBA titers against genetically diverse strains and that presents a lower risk of eliciting serum anti-FH autoantibodies.

In previous studies, two vaccine approaches enhanced SBA titers against meningococcal group B strains. The first was the use of native OMV (NOMV) from strains engineered to have genetically attenuated endotoxin activity (LpxL1 knockout [KO]) and FHbp overexpression (2, 12). With human peripheral blood mononuclear cells (PBMC), the penta-acylated lipopolysaccharide (LPS) in the NOMV produced from the LpxL1 KO mutant strain gave markedly decreased cytokine responses, which were similar to or lower than those elicited by a meningococcal dOMV vaccine that had been safely administered to tens of thousands of humans (2). Therefore, the NOMV-FHbp vaccines do not require treatment with detergents to decrease endotoxin activity (2). In mice, the NOMV-FHbp vaccine also stimulated higher serum anti-FHbp bactericidal antibody responses than recombinant FHbp vaccines (2, 13). The second approach used mutant FHbp antigens with low binding to FH (9, 14). The binding of FH to FHbp is specific for human and some nonhuman primate FH (15–17). Immunogenicity data from wild-type (WT) and human FH transgenic mice indicated that the binding of FH to FHbp decreases anti-FHbp SBA responses (9, 10). Further, immunization of human FH transgenic mice or infant macaques with recombinant mutant FHbp vaccines containing one or two amino acid substitutions that decreased FH binding resulted in increased SBA titers compared to those in control animals immunized with WT FHbp antigens that bound FH (9, 14, 18, 19).

In a previous study, we prepared a prototype NOMV vaccine lot with overproduced mutant FHbp with one amino acid substitution (R41S), which decreased FH binding by >100-fold compared to that of WT FHbp (13). In WT mice, the vaccine elicited up to 40-fold higher SBA responses than a control recombinant FHbp vaccine given alone or mixed with NOMV prepared from an FHbp KO strain (13). The mouse antibodies to the NOMV also had bactericidal activity against a gonococcal strain. While the findings were encouraging, there are some important limitations of the mouse model used in the study. First, although only mutant FHbp antigens were tested, the study was performed in WT mice whose mouse FH does not bind to WT FHbp, so the effect of any residual binding of human FH on SBA would not have been observed. Second, while the penta-acylated LPS in the NOMV-FHbp vaccine produced from the LpxL1 KO strain elicited markedly decreased endotoxic responses with human and nonhuman primate PBMC (2, 20), the mutant is reported to be a strong Toll-like receptor 4 (TLR4) agonist with mouse cells (21). Therefore, in mice the mutant NOMV-FHbp vaccine likely has an adjuvant effect stronger than that which would be expected in humans or nonhuman primates. Third, the mouse NOMV-FHbp immunogenicity study did not contain a licensed meningococcal serogroup B vaccine as a comparator. To address these issues, in the present study, we investigated the immunogenicity of the same lot of NOMV-FHbp vaccine used in the mouse study in an infant rhesus macaque model using animals whose macaque FH bound to FHbp similarly to human FH (16, 17). Since with macaque PBMC the NOMV containing penta-acylated LPS gave markedly decreased cytokine responses, similar to the low responses of humans (20), an adjuvant effect of the mutant LPS in macaques would be expected to be similar to that in humans. Finally, we included a control licensed meningococcal group B vaccine (MenB-4C) which contains recombinant WT FHbp that binds macaque FH.

## RESULTS

### Serum IgG anti-FHbp antibody responses.

**FIG 1:**
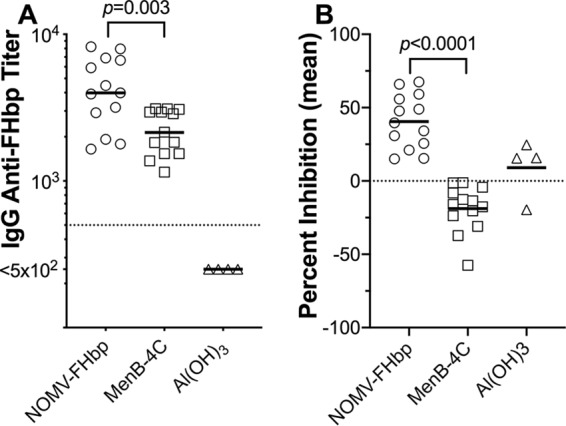
Macaque serum IgG anti-FHbp antibody responses 4 weeks after two vaccine doses. (A) Anti-FHbp antibody titers measured by ELISA using purified recombinant FHbp immobilized on the microtiter plate. The reciprocal geometric mean titers were 3,988 for the group vaccinated with NOMV-FHbp, 2,141 for the group vaccinated with MenB-4C, and <500 for the control group receiving the Al(OH)_3_ adjuvant (*P = *0.003 by a paired *t* test comparing the NOMV-FHbp and MenB-4C groups). (B) Ability of macaque serum antibodies to inhibit binding of FH to FHbp. Macaque sera were tested at a 1:50 dilution in the presence of 5 μg/ml of purified human FH. Antibodies to NOMV-FHbp inhibited the binding of FH to FHbp, whereas antibodies to MenB-4C enhanced the binding (*P < *0.0001 by a paired *t* test). The percent inhibition was calculated as described in Materials and Methods. Representative data from one of three experiments are shown.

The IgG anti-FHbp titers of all animals before vaccination were below the lowest serum dilution tested (<1:500). At 1 month after dose 2, the IgG titers remained below 1:500 in the adjuvant negative-control group and increased in both vaccine groups (Fig. 1A). The reciprocal geometric mean titer (GMT) was 2-fold higher for the NOMV-FHbp group than for the MenB-4C group (3,988 versus 2,141; *P = *0.003 by a paired *t* test).

Mouse FH does not bind to FHbp (9), and in previous studies, WT mice immunized with FHbp vaccines developed anti-FHbp antibodies that inhibited the binding of human FH to meningococci (9, 10). With less bound FH, there was less downregulation of complement activation, which resulted in higher bactericidal titers (22, 23). However, in human FH transgenic mice, the binding of FH to the WT vaccine antigen affected the anti-FHbp antibody repertoire and resulted in lower inhibition of FH binding (9, 10). Therefore, we measured the ability of sera from the immunized macaques to inhibit the binding of human FH to FHbp, as measured by an enzyme-linked immunosorbent assay (ELISA) (Fig. 1B). There was no significant inhibition or enhancement of binding of FH to FHbp in the four serum samples from macaques immunized with adjuvant alone (*P = *0.42 compared to a theoretical mean value of 0 by a one-sample *t* test). In contrast, the anti-FHbp antibodies elicited by the mutant FHbp antigen in the NOMV-FHbp vaccine inhibited the binding of FH to FHbp (mean inhibition, 40%; *P < *0.0001 by a one-sample *t* test), whereas the antibodies elicited by the WT FHbp antigen in MenB-4C enhanced the binding of FH (mean inhibition, −19%; *P = *0.001 by a one-sample *t* test). The difference between the respective mean inhibition values of the two vaccine groups was significant (*P < *0.0001 by a paired *t* test).

### Meningococcal SBA responses.

**FIG 2:**
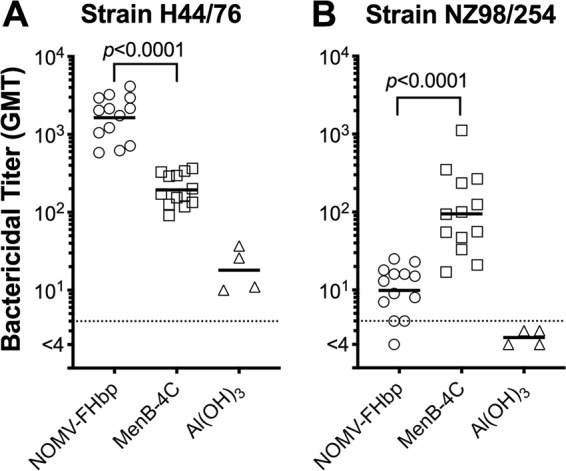
Serum bactericidal antibody (SBA) responses against strains with a PorA serosubtype matched to each of the two vaccines. (A) Strain H44/76, which is the parent strain of the mutant used to prepare NOMV-FHbp, has a matching PorA type (type 7, 16) and FHbp ID 1, which is matched to the FHbp variant in both vaccines. (B) Strain NZ98/254, which is used to prepare dOMV in MenB-4C, has a matching PorA type (type 7-2, 4) and relatively low expression of FHbp ID 14, which is 92% identical to FHbp in both vaccines. The geometric mean SBA titer against each strain was 8.4- to 9.5-fold higher for the respective vaccine with the matched PorA type than for the vaccine with the mismatched PorA type (*P < *0.0001 by paired *t* tests).

Each vaccine elicited higher reciprocal serum bactericidal antibody (SBA) GMTs against the respective test strain used to prepare the vaccine. For parent strain H44/76, which was used to prepare the mutant strain for the NOMV-FHbp vaccine and which matches the PorA and FHbp antigens in the NOMV-FHbp vaccine, the reciprocal GMT for macaques immunized with the NOMV-FHbp vaccine was 8.4-fold higher than that for macaques immunized with the MenB-4C vaccine (1,640 versus 194; *P < *0.0001; Fig. 2A). The reverse was observed for strain NZ98/254, which was used to prepare the dOMV in MenB-4C. This strain has a PorA that is mismatched to that in H44/76 and low expression of FHbp ID 14 (the FHbp identifier [ID] refers to specific amino acid sequence variants defined in a public database [https://pubmlst.org/neisseria/fHbp]), which is 91.8% identical to FHbp ID 1 in both vaccines. The reciprocal GMT was 9.5-fold higher in the MenB-4C group than in the NOMV-FHbp group (95 versus 10; *P < *0.0001; Fig. 2B).

**FIG 3:**
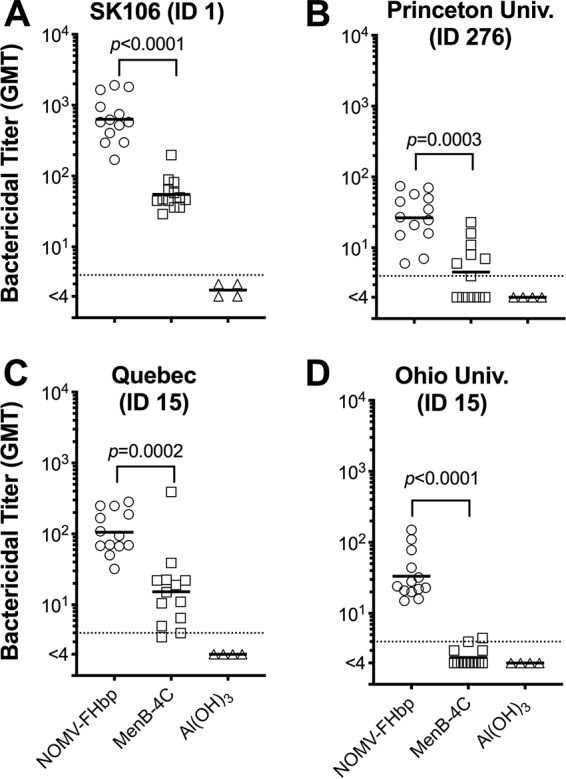
Serum bactericidal antibody (SBA) responses against meningococcal strains with PorA mismatched to the PorA in both vaccines and different FHbp sequence variants (Fig. 6). (A) Response against strain SK106 with FHbp ID 1 (identical to the FHbp in both vaccines) which was from an endemic case in Ohio. (B) Response against a strain with FHbp ID 276 (96% identical to the FHbp in both vaccines) from an outbreak at Princeton University, Princeton, NJ. (C) Response against a strain with FHbp ID 15 (88% identity to the FHbp in both vaccines) which is a case isolate recovered during hyperendemic disease in Quebec, Canada. (D) Response against another strain with FHbp ID 15 which was from an outbreak at Ohio University. The geometric mean bactericidal titers elicited by NOMV-FHbp were 5.8- to 13.8-fold higher than those elicited by MenB-4C (*P *≤* *0.0003 by paired *t* tests).

Against four additional meningococcal test strains with PorA mismatched to both vaccines, the NOMV-FHbp vaccine elicited 6- to 14-fold higher SBA GMTs than the MenB-4C vaccine. Strain SK106 expressed an FHbp ID 1 that matched the FHbp antigen in both vaccines (with the exception of the single R41S amino acid substitution in the NOMV-FHbp vaccine). Against this strain, the reciprocal GMT for the NOMV-FHbp vaccine group was 634 and that for the MenB-4C group was 55 (*P < *0.0001; Fig. 3A). For strains CH819 (Princeton University), CH860 (Quebec), and CH855 (Ohio University), which had FHbp in the same subfamily as both vaccines but whose sequences were not exactly matched for the FHbp antigens in the vaccines, the reciprocal GMTs for macaques immunized with the NOMV-FHbp vaccine and MenB-4C were 27 versus 5, respectively, for the Princeton University strain; 105 versus 15, respectively, for the Quebec strain; and 33 versus <4, respectively, for the Ohio University strain (*P *≤* *0.0003; Fig. 3B to D). Note that higher titers to NOMV-FHbp were observed, even though two of the test strains (CH855 and CH860) had high levels of expression of NHba, which is contained in MenB-4C, and two of the strains (SK106 and CH855) also expressed NadA (Table 1).

**TABLE 1:** Neisserial test strains used to measure serum bactericidal activity^*a*^

Strain	Description (reference)	Clonal complex^*b*^	PorA VR 1 and 2 sequence type^*c*^	FHbp ID^*d*^ (expression)^*e*^	NHba (expression)^*e*^	NadA gene presence (expression)^*e*^	Antigenic match
H44/76	Epidemic strain, Norway, parent of the mutant used to prepare NOMV-FHbp (51)	32	7,16	1 (++)	+/−	No	FHbp ID 1 and PorA (type 1.7,16) in NOMV-FHbp and FHbp ID 1 in MenB-4C
NZ98/254	Epidemic strain, New Zealand, used to prepare dOMV in MenB-4C (36)	41/44	7-2,4	14 (+/−)	+	No	PorA (type 4) in dOMV of MenB-4C
SK106	Endemic strain, Ohio (53)	32	19,15	1 (++)	ND	Yes (ND)	FHbp subfamily B in NOMV-FHbp and MenB-C
CH819	Outbreak, Princeton University, Princeton, NJ (54)	41/44	5,2-2	276 (+)	+	No	FHbp subfamily B in both vaccines
CH860	Hyperendemic strain, Quebec, Canada (56)	269	9,15-11	15 (++)	++	No	Subfamily B FHbp in both vaccines and NHba in MenB-4C
CH855	Outbreak strain, Ohio University (55)	269	22,2-9	15 (+)	++	Yes (++)	FHbp subfamily B in both vaccines and NadA and NHba in MenB-4C
FA1090	Isolate from disseminated gonococcal infection and first gonococcal genomic sequence (58)	NA^*f*^	Absent	NA (not surface expressed)	++	No	NHba in MenB-4C and NOMV-FHbp

a All strains were Neisseria meningitidis capsular group B, except for strain FA1090, which was N. gonorrhoeae.

b Based on multilocus sequence typing (59).

c PorA variable regions (VR) 1 and 2 (60).

d The FHbp identifier (ID) refers to specific amino acid sequence variants defined in a public database (https://pubmlst.org/neisseria/fHbp). See also Fig. 6.

e The expression of FHbp and NHba was measured by flow cytometry using mouse antiserum prepared to recombinant proteins that were matched to those in MenB-4C. NadA gene presence was determined by quantitative PCR (61). ND, not determined.

f NA, not available.

### Gonococcal serum bactericidal antibody responses.

In a previous study, 11 of 15 mice immunized with three doses of the NOMV-FHbp vaccine developed SBA titers of ≥1:5 against a gonococcal test strain (13). In the present study, only 3 of 13 macaques immunized with two doses of the same lot of the NOMV-FHbp vaccine, 1 of 13 immunized with MenB-4C, and 0 of 4 immunized with the aluminum hydroxide [Al(OH)_3_] adjuvant had gonococcal SBA responses (titers, ≥1:5; Fig. 4).

**FIG 4:**
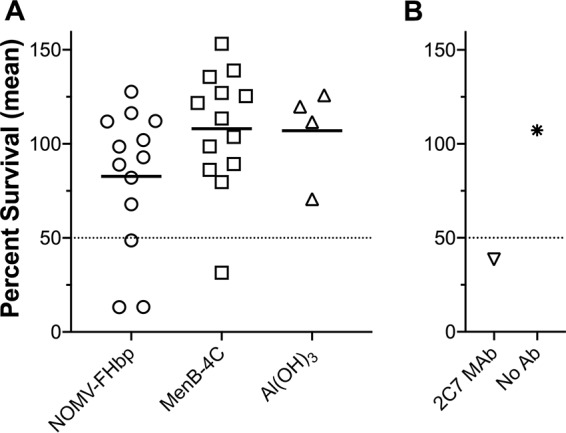
Serum bactericidal antibody responses against gonococcal strain FA1090. The *y* axes show the percent survival in the presence of human complement and macaque serum antibody. (A) Results for macaque sera. A positive SBA response was defined as ≤50% bacterial survival (which was found for 3/13 macaques in the NOMV-FHbp group versus 1/13 macaques in the MenB-4C group and 0/4 macaques in the aluminum hydroxide adjuvant control group) (*P = *0.18 comparing macaques receiving NOMV-FHbp and macaques receiving MenB-4C by Fisher’s exact test). (B) Results for control anti-gonococcal LPS monoclonal antibody (Ab) 2C7 (62).

### Anti-FH autoantibodies.

**FIG 5:**
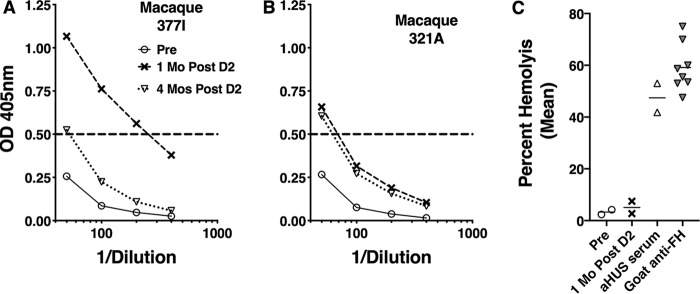
Serum anti-FH antibody response of macaques immunized with MenB-4C. (A and B) Results of anti-FH ELISA. Two of 13 macaques immunized with MenB-4C showed increases in anti-FH antibody at 1 month after vaccination. At 4 months, the titers in both animals remained elevated compared to the respective preimmunization titers. None of the 17 macaques immunized with NOMV-FHbp or Al(OH)_3_ adjuvant alone had elevated titers after immunization (OD_405_, <0.4 at a serum dilution of 1:50). (C) Serum FH functional activity measured by alternative pathway-mediated hemolysis of sheep red blood cells. The sera from the two macaques (macaques 377I and 321A) with elevated anti-FH antibody by ELISA had normal FH function, as evidenced by low hemolysis (see Materials and Methods). The positive human serum had deceased FH function associated with elevated anti-FH antibodies; the positive goat anti-FH serum was added (5%) to a human serum pool with normal FH function. Data are shown for 1:9 final dilutions of the test sera.

In previous studies in human FH transgenic mice and infant macaques immunized with MenB-4C, a few animals developed serum anti-FH antibodies after vaccination (10, 11). In the present study, 2 of 13 macaques immunized with MenB-4C showed significant increases in anti-FH antibody reactivity, which persisted above preimmunization levels for 4 months (Fig. 5). In contrast, none of the 13 macaques immunized with NOMV-FHbp or the 4 animals immunized with the Al(OH)_3_ adjuvant showed significant increases in serum anti-FH antibody (optical density at 405 nm [OD_405_], <0.40 at a serum dilution of 1:50).

Autoantibodies to FH can impair FH function (24, 25), which increases the risk of atypical hemolytic-uremic syndrome (aHUS). We therefore tested the sera from the two macaques with elevated anti-FH antibody titers (macaques 377I and 321A; Fig. 5A and B, respectively) for their ability to impair the functional activity of macaque FH to protect sheep red blood cells from alternative pathway (AP)-mediated hemolysis (26). In this assay, a low percentage of hemolysis implies the presence of fully functional serum FH, whereas more lysis indicates dysfunctional FH, which can result from the macaque autoantibodies. Pre- or postimmunization sera from macaques 377L and 321A did not impair FH function, even though the postimmune sera had elevated anti-FH antibody titers (Fig. 5C). In contrast, a normal human serum pool mixed with a goat antiserum to FH had impaired FH function, as did a human serum sample with anti-FH antibodies that was known to have impaired FH function.

## DISCUSSION

FHbp is relatively sparsely expressed by most meningococcal strains (27, 28), and NOMV vaccines prepared from WT strains elicited relatively low antibody responses to FHbp (29). However, in mice NOMV vaccines prepared from mutant meningococcal strains engineered to overproduce FHbp elicited higher SBA titers than recombinant FHbp vaccines or the recombinant protein antigens in the MenB-4C vaccine (2). In the present study, we compared the immunogenicity of a licensed meningococcal group B vaccine to that of a prototype meningococcal NOMV-FHbp vaccine with genetically attenuated endotoxin and an overexpressed FHbp mutant with low binding to human and macaque FH. The two vaccines differed in a number of other important respects: (i) MenB-4C contains dOMV, whereas our prototype vaccine contains NOMV, which preserves the lipoproteins in the latter vaccine; (ii) MenB-4C has a recombinant nonlipidated FHbp, whereas NOMV-FHbp has lipidated FHbp; (iii) MenB-4C has WT FHbp that binds macaque and human FH, whereas NOMV-FHbp has mutant FHbp with low binding to macaque and human FH; (iv) MenB-4C has two additional recombinant antigens, NHba and NadA; and (v) the dose of MenB-4C had a 12.5-fold larger amount of FHbp (∼25 μg in MenB-4C versus 2 μg in the NOMV-FHbp vaccine).

Despite the higher FHbp dose in the MenB-4C vaccine, the macaques immunized with NOMV-FHbp had 2-fold higher serum anti-FHbp antibody titers and the anti-FHbp antibody repertoire inhibited the binding of FH to FHbp, whereas the anti-FHbp antibodies elicited by MenB-4C increased the binding of FH. The ability of the serum anti-FHbp antibodies to inhibit the binding of FH may have contributed to the higher SBA titers elicited by NOMV-FHbp, particularly against the strains with relatively small amounts of surface-exposed FHbp and/or with sequence variants that did not exactly match the vaccine FHbp antigen. With less bound FH on the bacterial surface, there would be less FH downregulation of complement activation and higher SBA responses (22, 23). The basis for the higher IgG titers in the NOMV-FHbp group is incompletely understood, but they might have resulted from the lipidation of FHbp in the NOMV-FHbp vaccine rather than from the nonlipidated soluble antigen in the MenB-4C vaccine, the greater exposure of FHbp epitopes because the mutant FHbp does not bind FH, and/or the better preservation of native epitopes in NOMV. The basis for the difference in the antibody repertoire resulting in inhibition of FH binding by antibodies to the NOMV-FHbp vaccine likely involves the binding of FH to the WT FHbp antigen in MenB-4C but not to the mutant FHbp antigen in NOMV-FHbp, since similar differences were seen in a previous study of infant macaques immunized with a recombinant WT antigen or a mutant FHbp antigen with low FH binding (14).

The higher IgG anti-FHbp titers and the inhibition of FH binding in the NOMV-FHbp-vaccinated group appeared to be important for eliciting high SBA responses. Thus, the sera from macaques given the NOMV-FHbp vaccine had up to 14-fold higher geometric mean SBA titers than the sera from macaques given MenB-4C. Further, all 13 macaques immunized with NOMV-FHbp had SBA titers of ≥1:4 against the strains responsible for outbreaks at Ohio University and Princeton University, whereas 2 of 13 and 7 of 13 macaques given MenB-4C had SBA titers of ≥1:4 against these two strains, respectively (*P < *0.0001 and *P = *0.049, respectively, by Fisher’s exact test). In humans, SBA titers of ≥1:4 correlate with protection against developing meningococcal disease (30). Thus, the NOMV-FHbp vaccine not only stimulated higher overall SBA titers than MenB-4C but also protected against two virulent outbreak strains that were resistant to SBA elicited by the licensed vaccine.

In previous studies of mice immunized with NOMV vaccines with overexpressed WT FHbp, depletion of serum anti-FHbp antibodies removed the majority of SBA against strains with heterologous PorA to the vaccine (2, 18, 29). Further, with a few exceptions, mice immunized with control meningococcal NOMV vaccines prepared from FHbp knockout strains did not develop SBA (13, 31). The exceptional strains were mainly meningococci with PorA variable region (VR) sequence types matched to the vaccine. Thus, most of the broad SBA elicited by the NOMV-FHbp vaccines were directed at FHbp. Conceivably, in immunized adult humans who have been naturally exposed to neisserial antigens, SBA responses to NOMV-FHbp vaccines may be directed at other NOMV antigens, in addition to PorA, as was observed after dOMV vaccination (32). In the present study, strain NZ98/254, which is used to prepare the dOMV in MenB-4C, has a PorA VR sequence type mismatched to the NOMV-FHbp vaccine and low expression of FHbp. This strain, which has a PorA homologous to that in the dOMV in MenB-4C, was the only one of the six test strains against which SBA titers were higher in the animals immunized with MenB-4C than in the animals immunized with NOMV-FHbp.

Both the NOMV-FHbp and MenB-4C vaccines contain FHbp in subfamily B (ID 1). MenB-4C also contains two other recombinant antigens, NHba and NadA, which are responsible for the majority of SBA against strains with heterologous PorA and FHbp variants in subfamily A (33). In the present study, we did not test SBA responses against strains with FHbp subfamily A since our primary goal was to compare the breadth of the anti-FHbp SBA elicited by both vaccines. Mutants of subfamily A FHbp antigens with low FH binding have been described, and in human FH transgenic mice, these mutant antigens elicited higher SBA titers than the respective WT antigens (34, 35). To maximize the breadth of protection in humans, an NOMV-FHbp vaccine would need to include overexpressed mutant FHbp antigens from both subfamilies.

In New Zealand, a meningococcal dOMV vaccine referred to as MeNZB was prepared from group B strain NZ98/254 and used to control a persistent group B epidemic (36). Approximately 1 million persons ages 2 months to 20 years were vaccinated (37). Vaccination with MeNZB was estimated to be ∼70% effective in protecting against meningococcal disease caused by the epidemic meningococcal strain (37). Epidemiologic studies also suggested that MeNZB conferred partial protection against gonococcal disease (38). The dOMV component of the licensed MenB-4C vaccine is identical to that of MeNZB, and there has been interest in whether MenB-4C might also protect against gonococcal disease (39, 40). A recent study reported that humans immunized with MenB-4C developed serum antibodies that bind to gonococcal proteins (41). However, in our study of sera from adult humans immunized with MenB-4C or the second licensed group B vaccine, MenB-FHbp, we did not detect gonococcal SBA (13). In contrast, sera from the majority of mice immunized with three doses of the same NOMV-FHbp vaccine tested in the present nonhuman primate study had SBA against gonococcal strain FA1090 (13). In the present study, only 3 of 13 macaques in the NOMV-FHbp group had gonococcal SBA titers of ≥1:5 after immunization. In mice, the more prevalent SBA against the gonococcus could be related to the strong TLR4 agonist properties of the mutant penta-acylated LPS in NOMV-FHbp, which in humans (42) and macaques (20) is attenuated. Note that the data suggesting that MeNZB vaccination is protective against gonococcal disease are based largely on the findings of a study with a retrospective case-control study design, which is prone to unintentional bias from residual confounding variables. Such potential variables could have led to a false conclusion of protection, especially given the modest point estimate of effectiveness (31%). However, if there is protection against gonococcal disease conferred by MenB-4C, it does not appear to be dependent on SBA responses.

In the present study, two of the macaques vaccinated with MenB-4C developed serum autoantibodies to FH, which is consistent with our previous observations in human FH transgenic mice (10) and infant macaques (11). More recently, we also found serum anti-FH antibodies in postimmunization sera from a few human adults immunized with MenB-4C (K. Sharkey, P. T. Beernink, J. M. Langley, S. Gantt, C. Quach, C. Dold, Q. Liu, M. Galvan, and D. M. Granoff, submitted for publication). With only one exception, the human anti-FH antibodies did not impair serum FH function. Interpretation of the possible relationship between vaccination and impaired FH function in the exceptional subject postimmunization was limited by the lack of preimmunization serum from that subject.

Serum autoantibodies to FH are present in up to 8% of healthy adults without causing disease (43). However, anti-FH autoantibodies have been implicated in the pathogenesis of aHUS (44) and C3 glomerulopathies (C3G) (24). The underlying mechanism appears to be a decrease in FH function caused by the antibodies (24, 45), which results in less downregulation of complement activation. To date, there is no evidence that vaccination with MenB-4C or MenB-FHbp increases the risk of developing these diseases. While the anti-FH antibodies in the sera from the two immunized macaques in the present study did not impair FH function, the hemolytic assay used to measure FH function is mainly helpful for assessing the risk of disease in patients with aHUS. More sensitive assays are needed to assess the risk of C3G (24), but such assays were not available for investigation of the macaque sera. Although none of the macaques immunized with NOMV-FHbp or the aluminum hydroxide adjuvant alone developed anti-FH autoantibodies, the small sample sizes were insufficient to determine whether the risk of autoantibodies is actually higher in the MenB-4C-vaccinated group. If this is the case, the risk could theoretically be decreased through the use of mutant FHbp antigens with low FH binding.

## MATERIALS AND METHODS

### Infant rhesus macaques.

The macaques were born and housed at the California National Primate Research Center (University of California, Davis) in accordance with the American Association for Accreditation of Laboratory Animal Care standards and with strict adherence to the guidelines in the *Guide for the Care and Use of Laboratory Animals* (46). The infants were maintained in outdoor social housing with their dams and extended families. The study was approved by the Institutional Animal Care and Use Committee of the University of California, Davis. We collected serum samples from 107 macaques at 2 to 3 months of age and screened the sera for the binding of macaque FH to FHbp ID 1 by enzyme-linked immunosorbent assay (ELISA) as previously described (16). The exons encoding FH short consensus repeat domain 6 were amplified by PCR and subjected to DNA sequencing (16). Thirty macaques with high binding of FH to FHbp by ELISA and genotypes consistent with high binding (17) were enrolled in the immunogenicity study. Twenty-six macaques were vaccinated with a meningococcal vaccine (13 pairs, each matched for age, gender, and FH domain 6 genotype), and four were vaccinated with adjuvant alone and served as negative controls.

### Vaccines.

The preparation and characterization of the NOMV-FHbp vaccine have been described previously (13). In brief, the vaccine was prepared from a mutant strain of H44/76 with inactivation of the LpxL1 gene to attenuate endotoxin activity (47) and overexpression of FHbp ID 1 with one amino acid substitution (R41S). The mutation decreases the binding of human or macaque FH by more than 100-fold (9). NOMV-FHbp consisted of membrane blebs spontaneously released by the bacteria during growth in Frantz medium (48) and separated from bacteria by filtration (pore size, 0.2 μm; Millipore). Ultrafiltration (molecular weight cutoff, 100 kDa; Amicon) was used to concentrate the NOMV-FHbp. Based on the results of liquid chromatography-tandem mass spectrometry analysis, a 25-μg dose of the NOMV-FHbp vaccine contained as the most prominent proteins FHbp (2.0 μg), PorA (0.9 μg), PorB (3.8 μg), RmpM (2.7 μg), FetA (2.2 μg), and TbpA (0.9 μg) (13).

The MenB-4C vaccine was purchased commercially. One human dose contains 25 μg of dOMV prepared from group B strain NZ98/254 and 50 μg each of three recombinant proteins, FHbp (ID 1) and neisserial heparin binding antigen (peptide 2), each purified as fusion proteins (49), and neisserial adhesin A (peptide 8) (https://www.fda.gov/downloads/biologicsbloodvaccines/vaccines/approvedproducts/ucm431447.pdf, accessed 15 April 2019).

### Macaque immunogenicity.

A group of 13 infant macaques received 25 μg of NOMV-FHbp (13), which was adsorbed with 1.5 mg of aluminum hydroxide [Al(OH)_3_] as the adjuvant. The dose of NOMV-FHbp, which was measured by a DC protein assay (Bio-Rad), and the amount of Al(OH)_3_ were chosen to match the respective amounts of dOMV and Al(OH)_3_ in a human dose of MenB-4C. As positive controls, 13 macaques each received a human dose of MenB-4C. As negative controls, four macaques received Al(OH)_3_ adjuvant alone. At ages 3 to 4 months, the animals were vaccinated intramuscularly with a 0.5-ml dose divided into two 0.25-ml aliquots, which were given as separate injections in each leg. A second dose was given 6 weeks later, followed by blood collection 4 weeks after the second dose.

### Serum IgG anti-FHbp antibody responses.

Serum IgG anti-FHbp titers were measured by ELISA (50). Briefly, recombinant FHbp ID 1 (2 μg/ml in phosphate-buffered saline) was added to the wells of a microtiter plate (Immulon 2 HB; Thermo Fisher), and the plate was incubated overnight at 4°C. After blocking, 5-fold serial dilutions of macaque serum starting at 1:500 were added and the plate was incubated for 1 h at room temperature. Bound macaque IgG was detected with goat anti-human IgG (Fc specific) conjugated to alkaline phosphatase (1:5,000; Sigma-Aldrich), which cross-reacts with macaque IgG. After 30 min of development with *para*-nitrophenyl phosphate substrate (1 mg/ml; Sigma), the optical density (OD) at 405 nm was measured in a plate reader.

### Ability of serum antibodies to inhibit binding of FH to FHbp.

We used an ELISA to measure the ability of macaque serum antibodies to inhibit the binding of factor H to immobilized FHbp as previously described for mouse serum antibodies (10). Microtiter plates were coated with FHbp ID 1 as described above. After blocking, 3-fold serial dilutions of the macaque serum samples starting at 1:50 were premixed with a fixed concentration of human FH (5 μg/ml; Complement Technologies) and added to the wells of the plate, which was incubated at room temperature for 1 h. After washing, bound human FH was detected with polyclonal sheep anti-human FH antibody (1:7,000; Abcam), followed by donkey anti-sheep IgG conjugated to alkaline phosphatase (1:5,000; Sigma-Aldrich). Normal macaque serum (1% [vol/vol]) was added to the sheep antiserum as a blocking reagent to prevent the binding of the secondary antibody to the macaque IgG bound to FHbp. The percent inhibition was calculated as [1 − (*A_X_*/*A*_0_)] × 100 (one minus the ratio of FH binding to immobilized FHbp in the presence [*A_X_*] versus the absence [*A*_0_] of serum antibody multiplied by 100).

### Meningococcal and gonococcal strains.

We measured the SBA responses against six meningococcal capsular group B strains and one strain of Neisseria gonorrhoeae (Table 1). H44/76 is a meningococcal case isolate from an epidemic in Norway (51); this strain was used to construct the mutant strain used to prepare the NOMV-FHbp vaccine and therefore is matched to PorA and FHbp ID 1 (except for the one amino acid substitution in the NOMV-FHbp vaccine). H44/76 is also matched to FHbp ID 1 in the MenB-4C vaccine (49, 52). Strain NZ98/254 is a case isolate from an epidemic in New Zealand (36); this strain is used to produce the dOMV component of MenB-4C and therefore is matched to PorA for that vaccine. NZ98/254 also expresses a relatively small amount of FHbp ID 14 (Table 1), which is 91.8% identical to FHbp ID 1 in both vaccines.

**FIG 6:**
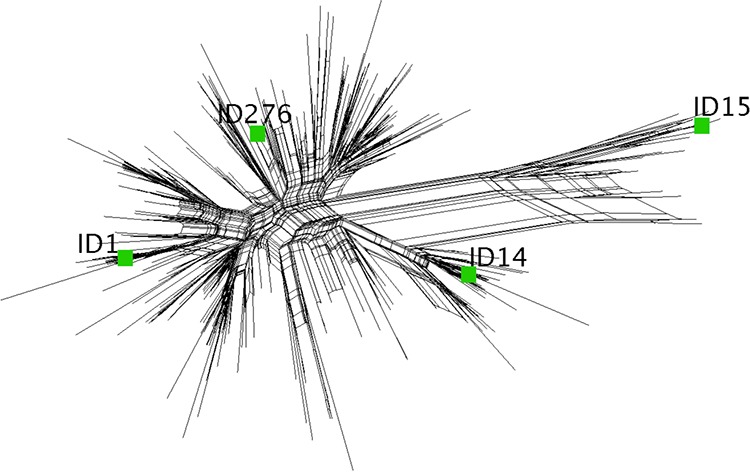
A SplitsTree representation of amino acid sequence diversity within FHbp subfamily B is shown for 325 FHbp sequences. The MenB-4C vaccine contains recombinant FHbp ID 1; the NOMV-FHbp vaccine contains overexpressed mutant FHbp ID 1 with one amino acid substitution (R41S). Two of the test strains used to measure SBA expressed FHbp ID 1 (Table 1). The relationships of the FHbp sequences with ID 14, 15, or 276, expressed by the other four test strains (Table 1) used to measure SBA, are shown. The figure was generated with the SplitsTree (version 4) program (63).

We also tested SBA against four additional meningococcal strains with PorA mismatched to that of both vaccines. Strain SK106 is a case isolate from a patient hospitalized in Ohio in 2003 (53) and is matched to FHbp ID 1 in both vaccines; the NadA gene is present, and the NHba allele has not been determined. Strains CH819, CH855, and CH860 are invasive case isolates from outbreaks at Princeton University (8, 54), at Ohio University (6, 55), and in the province of Quebec, Canada (56), respectively. CH819 expresses FHbp ID 276, which has 95.7% amino acid sequence identity to FHbp ID 1 in both vaccines. CH855 and CH860 express FHbp ID 15, which has 87.8% identity to FHbp ID 1; both strains also express NHba with cross-reactivity with the NHba antigen in MenB-4C (Table 1). CH855 also is a high expresser of NadA with cross-reactivity to NadA in MenB-4C. The FHbp sequence diversity is shown in a SplitsTree representation (Fig. 6). We also used gonococcal strain FA1090 to measure SBA since, in our previous study, mice immunized with the NOMV-FHbp developed SBA against this strain (13). This strain is also reported to express a number of surface-exposed antigens in MenB-4C (41), including NHba, which has 69% amino acid sequence identity with the recombinant NHba in MenB-4C.

### Serum bactericidal antibody assay.

For the meningococcal SBA assay, the bacteria were grown to mid-log phase in Frantz medium (48) supplemented with 4 mM d,l-lactate (Sigma-Aldrich) and 2 mM cytidine 5′-monophospho-*N*-acetyl-neuraminic acid (Carbosynth) to increase the sialylation of LPS (57). To inactivate endogenous complement, the test sera were heated for 30 min at 56°C. The exogenous human complement was a commercial serum pool depleted of IgG and IgM antibodies (Pel-Freez). SBA titers were assigned as the dilution resulting in a 50% decrease in the number of CFU of bacteria compared with the number of CFU of bacteria incubated for 60 min with negative-control sera and complement. For the gonococcal SBA assay, heat-inactivated sera were tested with strain FA1090 at a 1:5 dilution with 20% exogenous pooled normal human serum as the complement source.

### Anti-FH antibody.

Anti-FH antibody reactivity was measured by ELISA. Microtiter plates (Immulon 2HB; Thermo Scientific) were coated with purified human FH (2 μg/ml; Complement Technologies) and incubated overnight at 4°C. After washing, 2-fold serial dilutions of macaque sera starting at 1:50 were added, and bound macaque IgG was detected with goat anti-human IgG conjugated to alkaline phosphatase (1:5,000; Sigma-Aldrich), which cross-reacts with macaque IgG. To determine the effect of the anti-FH antibody on FH function, we used a hemolytic assay that measures the ability of serum FH to protect sheep red blood cells from alternative pathway-mediated hemolysis. In brief, serial dilutions of the test serum were performed with gelatin Veronal buffer (Complement Technologies) with added 10 mM EGTA and 7 mM MgCl_2_. Fifty microliters of diluted test sera (1:3) was mixed with 50 μl of sheep erythrocytes (Colorado Serum Company) and 50 μl of a 1:10 dilution of normal human serum that had been depleted of FH and factor D (Complement Technologies). After incubation at 37°C for 60 min, the reaction was stopped by the addition of 2 ml of cold saline, and hemolysis was calculated by measuring the amount of hemoglobin released into the supernatant based on the OD_415_. As positive controls, we included a normal human serum pool, tested in parallel, that had been mixed with a goat antiserum to human FH that blocks FH function (5% [vol/vol]; catalog number A312; Quidel) and a human serum sample with anti-FH autoantibody and impaired FH function.

### Statistical analyses.

IgG and SBA titers below the limit of detection were assigned half the value of the lowest dilution tested. Statistical tests were performed on log_10_-transformed values. Paired Student’s *t* tests were used to compare the geometric means between the experimental and positive-control vaccine groups. Fisher’s exact test was used to compare the numbers of animals positive or negative for SBA or for anti-FH antibody. All statistical tests were two-tailed, and *P* values of ≤0.05 were considered signiﬁcant.
